# Supplementary material for: Inhibition mediated by group III metabotropic glutamate receptors regulates habenula activity and defensive behaviors
Source: Nat Commun. 2025 Aug 5;16:7187. doi: 10.1038/s41467-025-62115-z (PMC12325729; doi:10.1038/s41467-025-62115-z)
Supplement: Supplementary file 1 — Supplementary Information [file 41467_2025_62115_MOESM1_ESM.pdf]

Supplementary Material

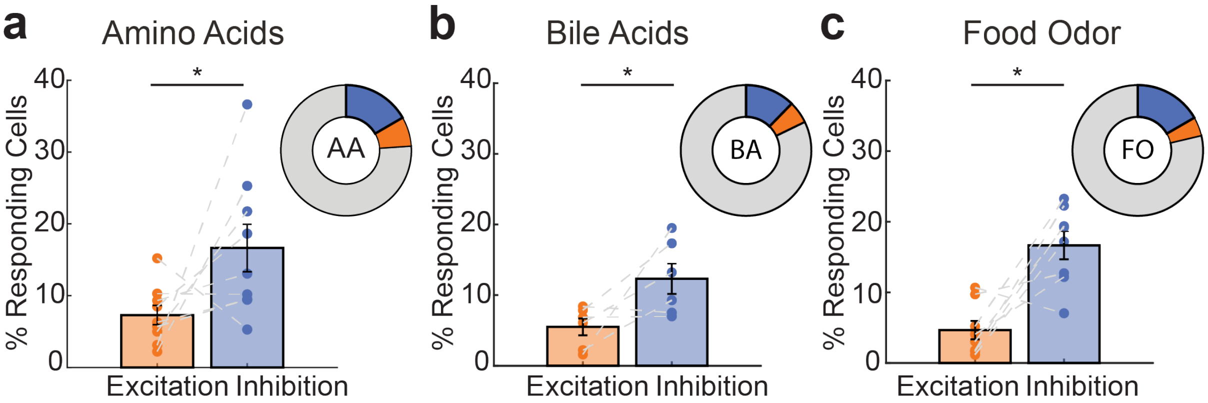

Supplementary Figure 1: Habenular neurons respond to different odors with both excitation and inhibition. Percentage of excited and inhibited cells in habenula upon amino acids (**a**, n=9 fish, Alanine, Phenylalanine, Methionine, Histidine, Cysteine, Arginine, Glutamic acid at 10^{-4} M, *p=0.0273), bile acids (**b**, n=6 fish, taurocholic acid, taurodeoxycholic acid $5x10^{-4}$ M, *p=0.0469) and food odor (**c**, n=8 fish, *p=0.0117) stimulations (one-sided Wilcoxon signed-rank test). Neurons with responses 2 STD above baseline are excited (orange), 1 STD below baseline are inhibited (blue). AA: amino acids, BA: bile acids, FO: food odor. Error bars represent mean +/-SEM. Scattered dots represent individual fish. Source data are provided as a Source Data file.

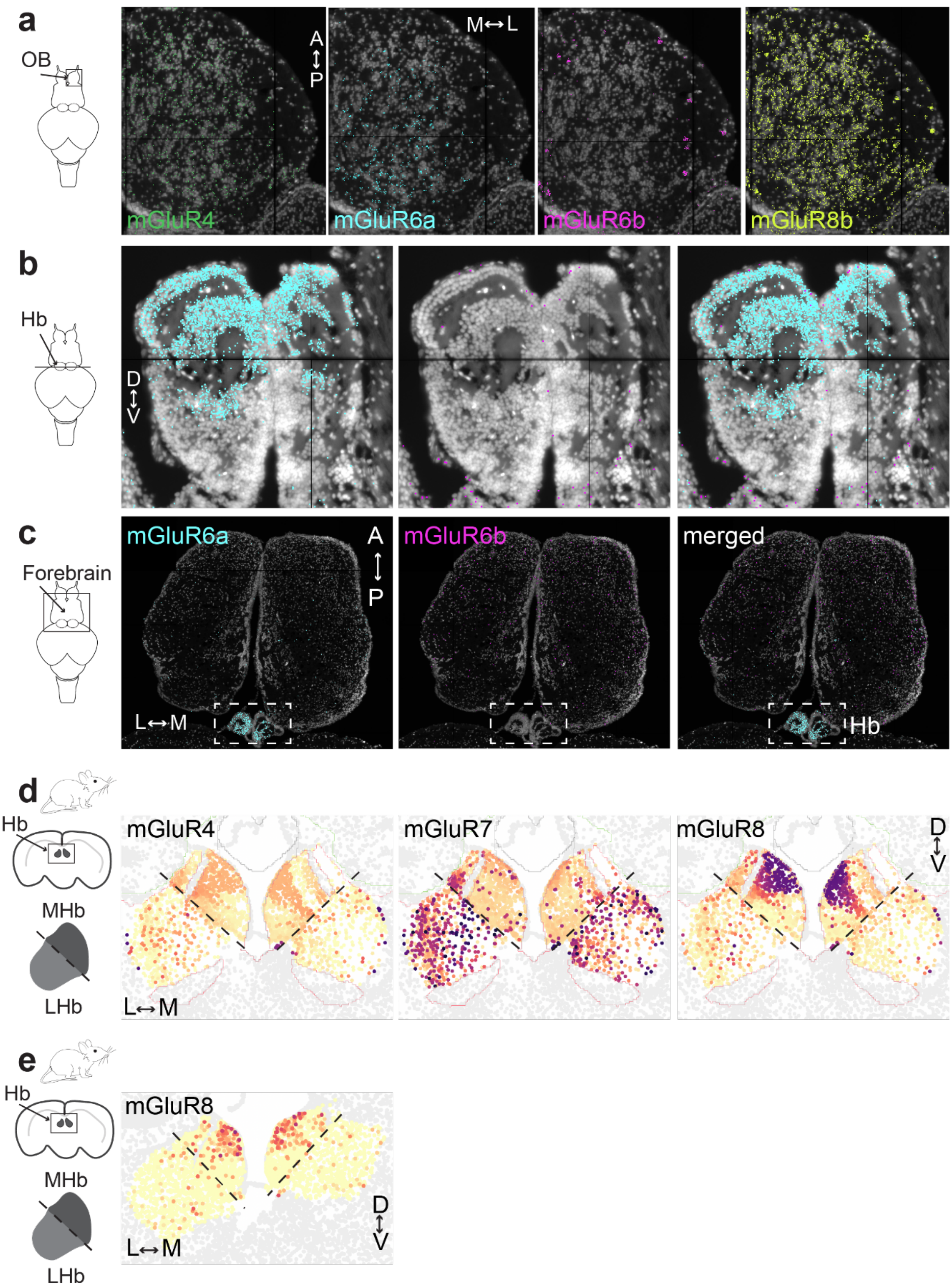

Supplementary Figure 2: Group III mGluR expression in the zebrafish olfactory bulb as well as zebrafish and mouse habenula. (**a**) High-resolution spatial transcriptome of adult zebrafish olfactory bulb (n=2 fish) illustrating the expressions of mGluR4 (green), mGluR6a (cyan), mGluR6b (pink) and mGluR8b (yellow).(**b, c**) High-resolution spatial transcriptomics for mGluR6a (cyan), and mGluR6b (pink) in adult zebrafish habenula (**c**, coronal section), and

dorsal forebrain (**d**, horizontal section), along the dorsal-ventral (DV), anterior-posterior (AP) and medial-lateral (ML) axes. (n=2 fish) (**d**) Coronal section of mouse habenula displaying the imputed spatial transcriptomics for mGluR4 (right), mGluR7 (middle) and mGluR8 (left). Darker colors represent higher expression. The dashed lines indicate the separation between medial (MHb) and lateral (LHb) Hb. Data are adapted from Allen Institute for Brain Science – Brain Knowledge Platform (**ABC Atlas**) (**e**) Spatial transcriptome data of mGluR8 expression in the mouse habenula. Darker colors represent higher expression. Data is adapted from Allen Institute for Brain Science – Brain Knowledge Platform (**ABC Atlas**). OB: olfactory bulb, Hb: habenula, LHb: lateral habenula, MHb, medial habenula, D: dorsal, V: ventral, L:lateral, M:medial, A:anterior, P:posterior.

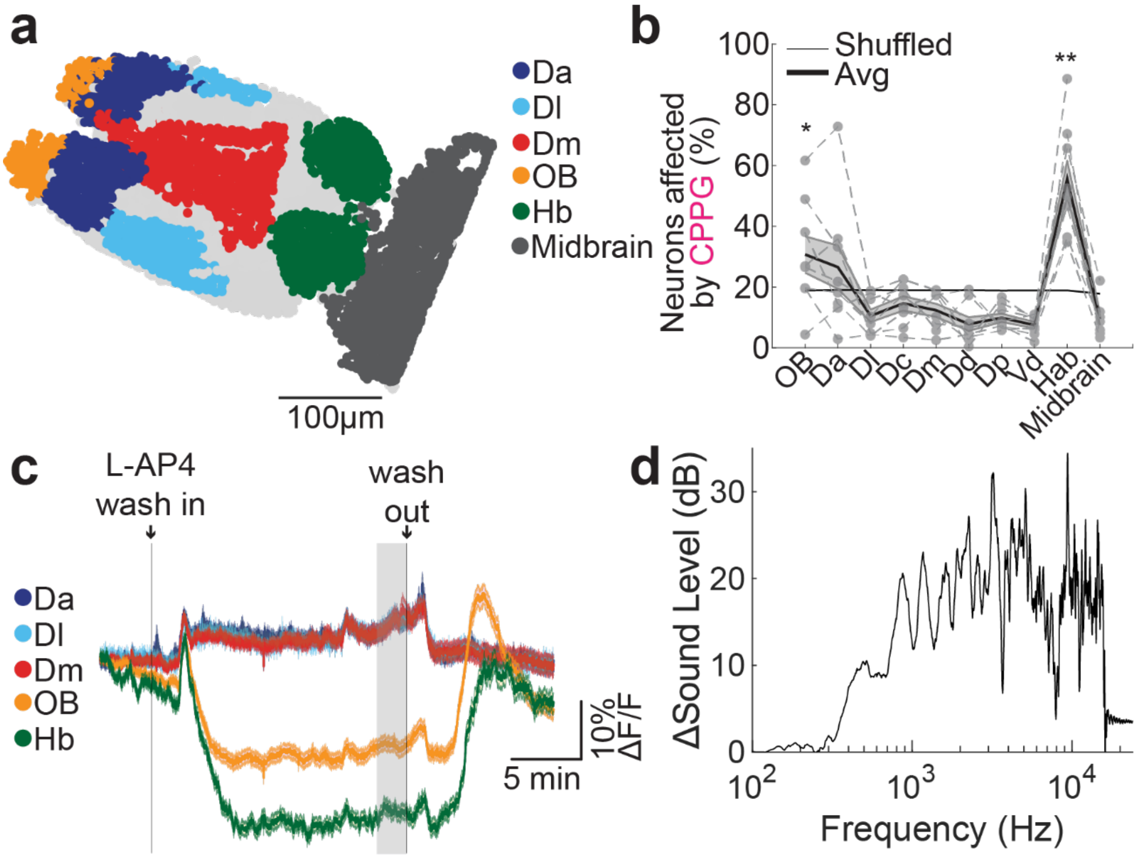

Supplementary Figure 3: Pharmacological targeting of group III mGluRs in midbrain and ventral habenula neurons (**a**) 3D reconstructions of neurons in juvenile T*g(elavl3:GCaMP6s-nuclear)* zebrafish forebrain color-coded by anatomically delineated forebrain regions as well as midbrain neurons (Dorso-anterior (Da): blue, Dorso-lateral (Dl): cyan, Dorso-medial (Dm): red, olfactory bulb (OB): yellow, habenula (Hb): green, midbrain: dark grey). Scale bar indicates 100 μm. (**b**) Percentage of neurons in anatomically identified regions that are affected by CPPG (300 μM). Affected means that during a 5 min drug period (shaded grey in Fig. 3**b**) neuronal calcium signals were 2STD above the baseline before drug wash-in. Scatters corresponding to individual fish are connected with dashed lines. Average is indicated as the thick black line; shadow presents +/-SEM. Grey line represents the shuffle distribution. Neurons in the olfactory bulb and the Hb are significantly more affected compared to shuffle distribution. Midbrain neurons are not significantly affected by CPPG. (CPPG n=8 fish, OB *p=0.0391, Hb **p=0.0039, rest is n.s. one-sided Wilcoxon signed-rank test). Dc:dorso-central, Dp:dorso-posterior, Vd:ventral-dorsal.(**c**) Average time courses of neuronal calcium signals from anatomically identified forebrain regions in response to bath application of L-AP4 (0.1 μM) in example fish. Shadow of the average traces represents +/-SEM. Grey lines indicate the wash-in and wash-out time points. Grey shaded area corresponds to the drug period used for calculations of affected neurons (see Fig.3 **d**). (**d**) Amplitude (dB) and frequency (Hz) of vibration stimuli. Source data are provided as a Source Data file.

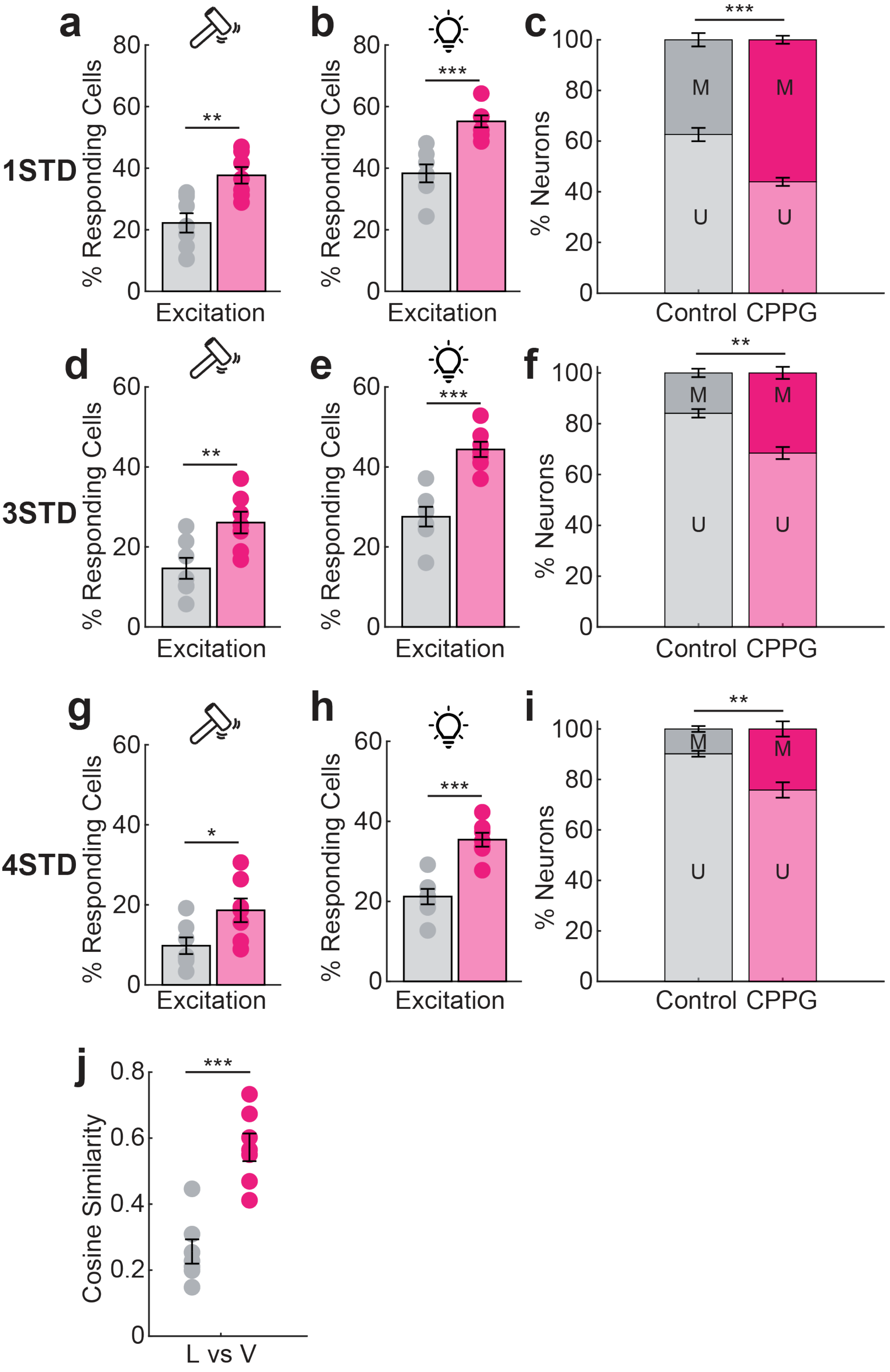

a
b
c
1STD
% Responding Cells
Excitation
% Neurons
Control CPPG
M
U
***
**
d
e
f
3STD
g
h
i
4STD
*
j
Cosine Similarity
L vs V

Supplementary Figure 4: Pharmacological blocking of group III mGluRs amplifies the magnitude and reduces the selectivity of sensory responses of habenular neurons, reanalyzed based on different response thresholds. (**ab, de, gh**) Percentage of excited habenula neurons for control (grey) or CPPG-injected (pink) fish in response to mechanical vibrations (**a,d,g**) or light (**b,e,h**) stimulation using different thresholds: To count as excited a neuron's response need to exceed a threshold of 1STD(**ab**), 3STD(**de**) or 4STD(**gh**) plus the mean during the baseline duration. Note that for all the different thresholds, CPPG-injected fish show significantly more excited cells than control for both vibration and light (Control n=7 fish, CPPG n=7 fish, 1std: vibration **p=0.0020, light ***p=0.0003; 3std: vibration **p=0.0084, light ***p=0.0006; 4std: vibration *p=0.0189, light ***p=0.0006, one-sided Wilcoxon rank sum test). (C,F,I) Percentage of unimodal (U) habenula neurons that respond exclusively to either light or vibrations versus multimodal (M) neurons responding to both light and vibrations for the different responding thresholds: 1std (**c**), 3STD (**f**) and 4STD (**i**). Note that for all the different thresholds, significantly less neurons in the CPPG-injected fish are selective for one of the two stimulus modalities (unimodal), instead more cells are multimodal (Control n=7 fish, CPPG n=7 fish, 1std: ***p=0.0003; 3std: **p=0.0012; 4std: **p=0.0020, one-sided Wilcoxon rank sum test). (**j**) Cosine similarity of multi-neuronal response vectors in the habenula for mechanical vibrations and light. Sensory responses in the CPPG-injected fish are significantly more similar to each other compared to control fish (Control n = 7, CPPG n = 7, ***p=0.0006, one-sided Wilcoxon rank sum test). Error bars represent mean +/-SEM. Scattered dots represent individual fish. Source data are provided as a Source Data file.

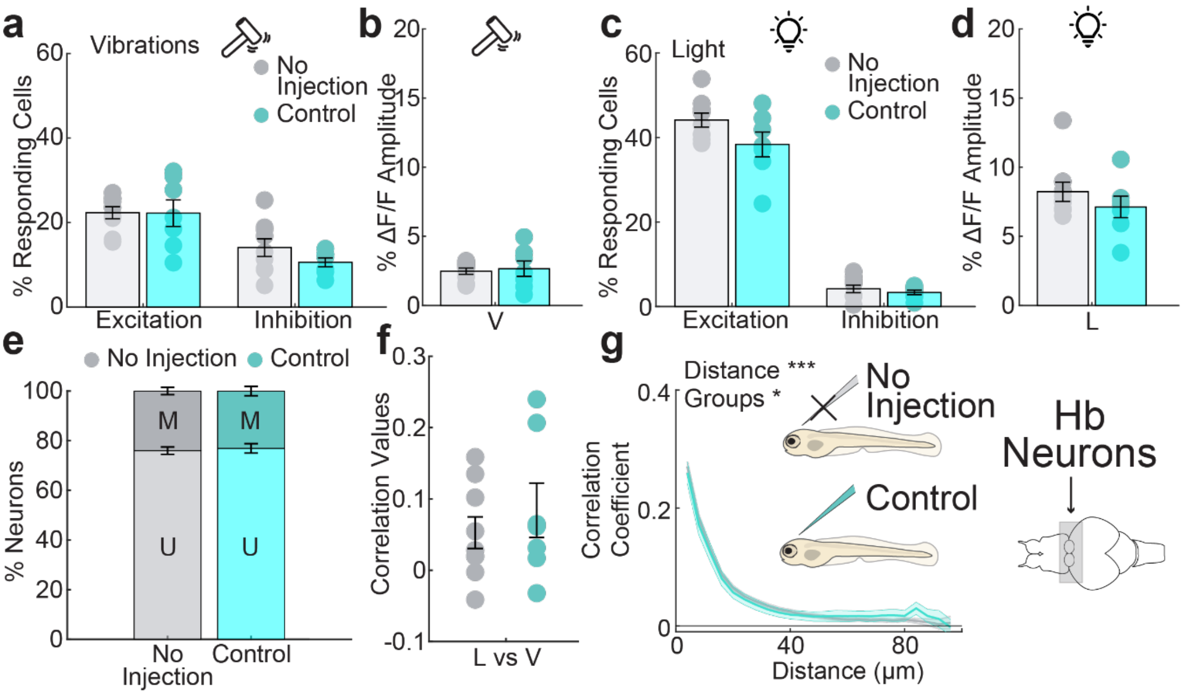

Supplementary Figure 5: Sensory responses in non-injected and control-injected zebrafish. Larval *Tg(elavl3:GCaMP6s)* zebrafish recorded by two-photon calcium imaging were presented with mechanical vibrations and light. (**a, c**) Percentage of excited (2 STD above baseline) or inhibited (2 STD below baseline) habenula (Hb) neurons for no injected fish (grey) or control-injected (cyan) fish in response to mechanical vibrations (**a**) or light (**c**) stimulation. Note that there is no difference between the groups. (No Injection n=9, Control n=7, p is n.s. one-sided Wilcoxon rank sum test). (**b, d**) Average ΔF/F amplitude (%) during the response period of all neurons in habenula per fish. (No Injection n=9, Control n=7, p is n.s. one-sided Wilcoxon rank sum test). (**e**) Percentage of unimodal (U) habenula neurons that respond exclusively to either light or vibrations versus multimodal (M) neurons responding to both light and vibrations. Note that there is no difference between the groups. (No Injection n=9, Control n=7, p is n.s. one-sided Wilcoxon rank sum test). (**f**) Pearson's correlation of multi-neuronal response vectors in the habenula for mechanical vibrations and light. Note that there is no difference between the groups. (No Injection n=9, Control n=7, p is n.s. one-sided Wilcoxon rank sum test). (**g**) Pairwise Pearson's correlation of habenula neurons during spontaneous activity as a function of distance (μm) between each neuron pair in no injection fish (grey) versus control-injected fish (cyan) (No Injection n=18 Hb hemispheres in 9 fish, Control n=14 Hb hemispheres in 7 fish, ANOVA-n displayed significance over distances ***p=8.7*10^-13 and over treatment groups *p=0.0268). Shadow represents +/-SEM. Schematic illustration indicates that data is from habenula neurons. Error bars represent mean +/-SEM. Scattered dots represent individual fish. Source data are provided as a Source Data file.

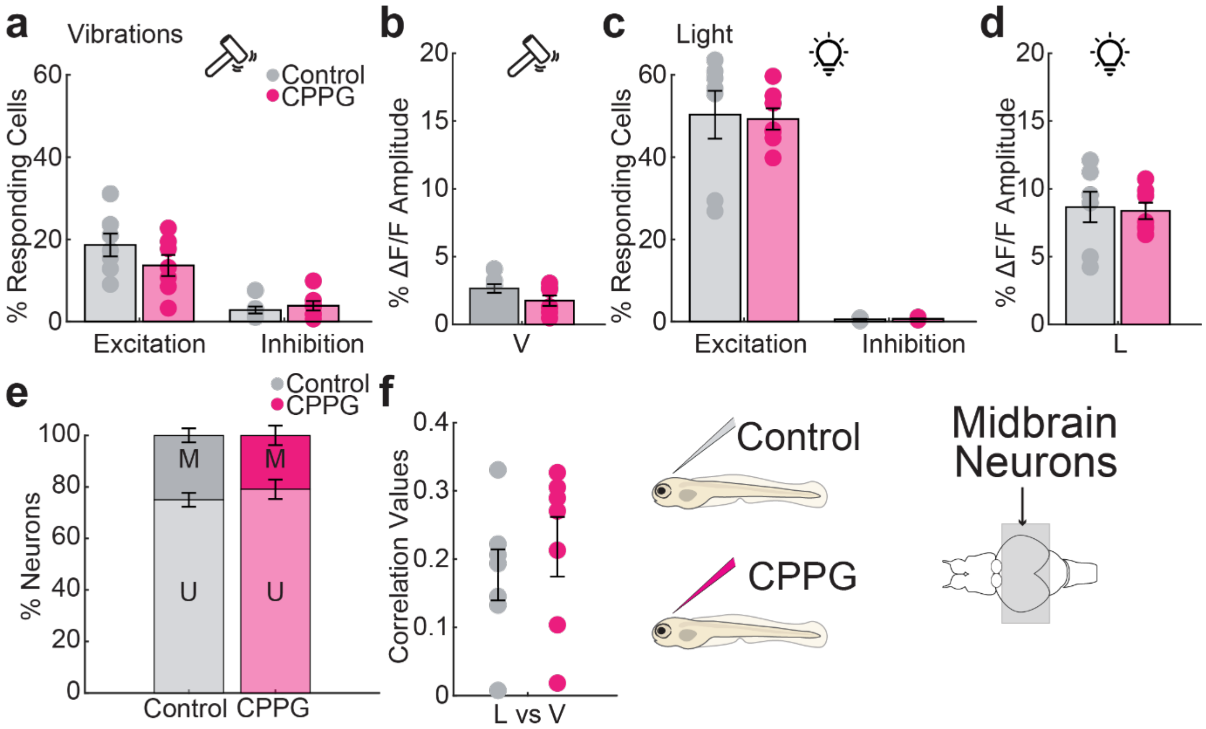

Supplementary Figure 6: Sensory responses midbrain neurons in control-injected and CPPG-injected zebrafish. Larval *Tg(elavl3:GCaMP6s)* zebrafish recorded by two-photon calcium imaging were presented with mechanical vibrations and light. (**a, c**) Percentage of excited (2 STD above baseline) or inhibited (2 STD below baseline) midbrain neurons for control-injected fish (grey) CPPG-injected (5 mM, pink) fish in response to mechanical vibrations (**a**) or light (**c**) stimulation. Note that there is no difference between the groups (Control n=7 fish, CPPG n=7 fish, p is n.s. one-sided Wilcoxon rank sum test). (**b, d**) Average ΔF/F amplitude (%) during the response period of all neurons in the midbrain per fish for vibration (**b**) and light (**d**) (Control n=7 fish , CPPG n=7 fish, p is n.s. one-sided Wilcoxon rank sum test). (**e**) Percentage of unimodal (U) midbrain neurons that respond exclusively to either light or vibrations versus multimodal (M) neurons responding to both light and vibrations. Note that there is no difference between the groups (Control n=7 fish, CPPG n=7 fish, p is n.s. one-sided Wilcoxon rank sum test). (**f**) Pearson's correlation of multi-neuronal response vectors in the midbrain for mechanical vibrations and light. There is no difference between the groups (Control n=7 fish, CPPG n=7 fish, p is n.s. one-sided Wilcoxon rank sum test). Schematic illustration indicates that data is from midbrain neurons. Error bars represent mean +/-SEM. Scattered dots represent individual fish. Source data are provided as a Source Data file.

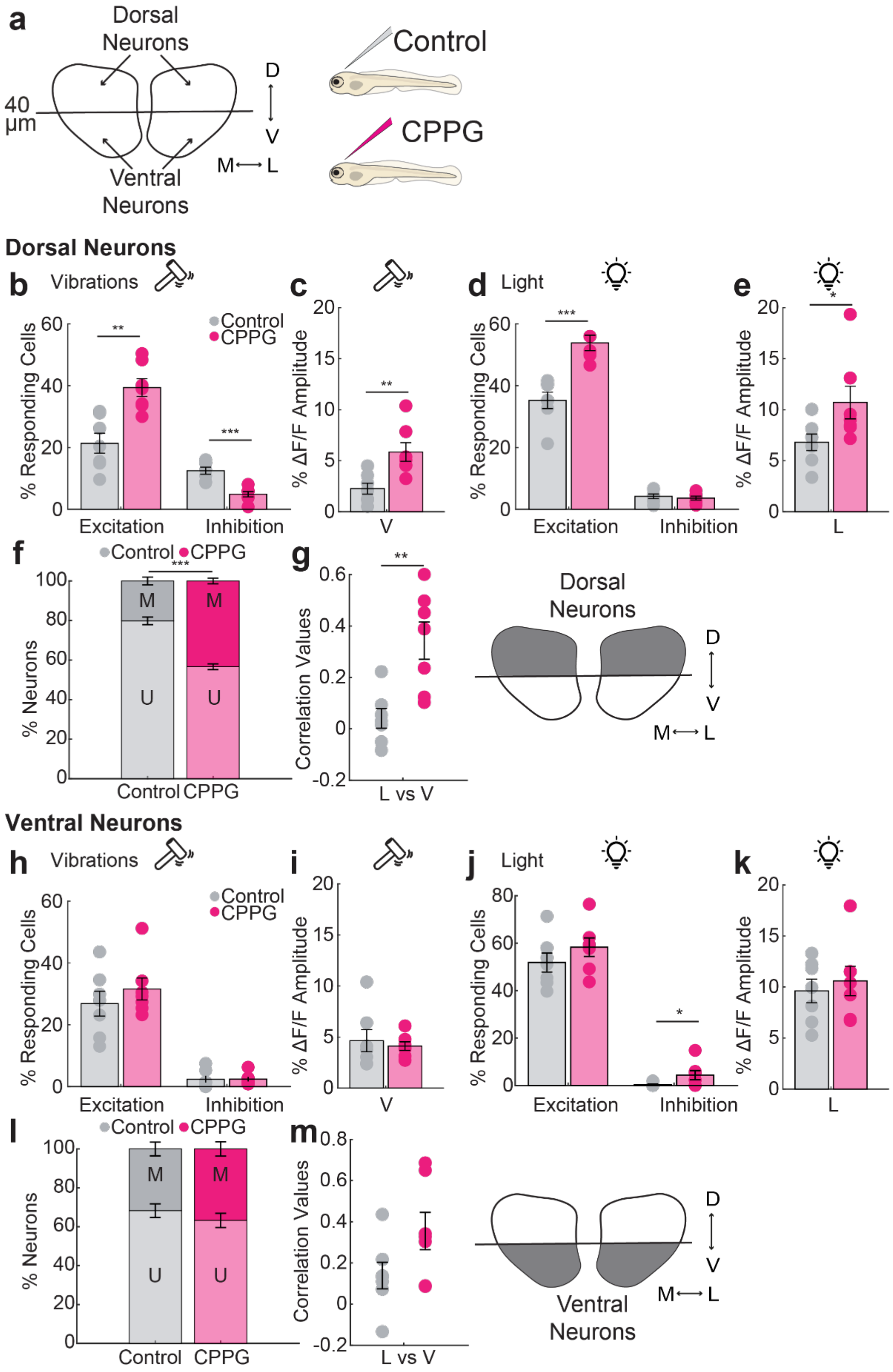

Supplementary Figure 7: Sensory responses in dorsal versus ventral habenula in control-injected and CPPG-injected zebrafish. Larval *Tg(elavl3:GCaMP6s)* zebrafish recorded by two-photon calcium imaging were presented with mechanical vibrations and light. (**a**) Scheme indicating how the habenula was split into dorsal vs ventral zones. The dorsal-

ventral position of each neuron was normalized. Neurons located in top 40 μm are dorsal (**b-g**) and below 40 μm are ventral (**h-l**). (**b,d,h,j**) Percentage of excited (2 STD above baseline) or inhibited (1 STD below baseline) habenula neurons for control-injected fish (grey) CPPG-injected (5 mM, pink) fish in response to mechanical vibrations (**b, h**) or light (**d, j**) stimulation. Note that there is a significant difference between the groups for the excited dorsal habenula neurons (**b,d**), but not for the ventral ones (**h,j**). There are significantly more inhibited neurons to light stimulation for the ventral habenula neurons (Control n=7 fish, CPPG n=7 fish, dorsal: vibration: excitation **p=0.0012, inhibition ***p=0.0003; light: excitation ***p=0.0003, inhibition p is n.s., ventral p is n.s., one-sided Wilcoxon rank sum test). (**c,e,i,k**) Average ΔF/F amplitude (%) during the response period of all habenular neurons in each fish. Note that there is a significant difference for the dorsal habenular neurons (**c,e**), but not for the ventral ones (**i,k**) (Control n=7 fish, CPPG n=7 fish, dorsal: vibration: **p=0.0012; light: *p=0.0189, ventral p is n.s., one-sided Wilcoxon rank sum test). (**f,l**) Percentage of unimodal (U) habenular neurons that respond exclusively to either light or vibrations versus multimodal (M) neurons responding to both light and vibrations. Note that there is a significant difference for the dorsal habenula neurons (**f**), but not the ventral ones (**l**) (Control n=7 fish, CPPG n=7 fish, dorsal: ***p=0.0003; ventral p is n.s., one-sided Wilcoxon rank sum test). (**g,m**) Pearson's correlation of multi-neuronal response vectors in the habenula for mechanical vibrations and light. Note that there is a significant difference for the dorsal habenula neurons (**g**), but not the ventral ones (**m**). (Control n=7 fish, CPPG n=7 fish, dorsal: **p=0.0035; ventral p is n.s., one-sided Wilcoxon rank sum test). Schemes indicate that data is either from the dorsal (**g**, right) or ventral habenula neurons (**m**, right). Error bars represent mean +/-SEM. Scattered dots represent individual fish. Source data are provided as a Source Data file.

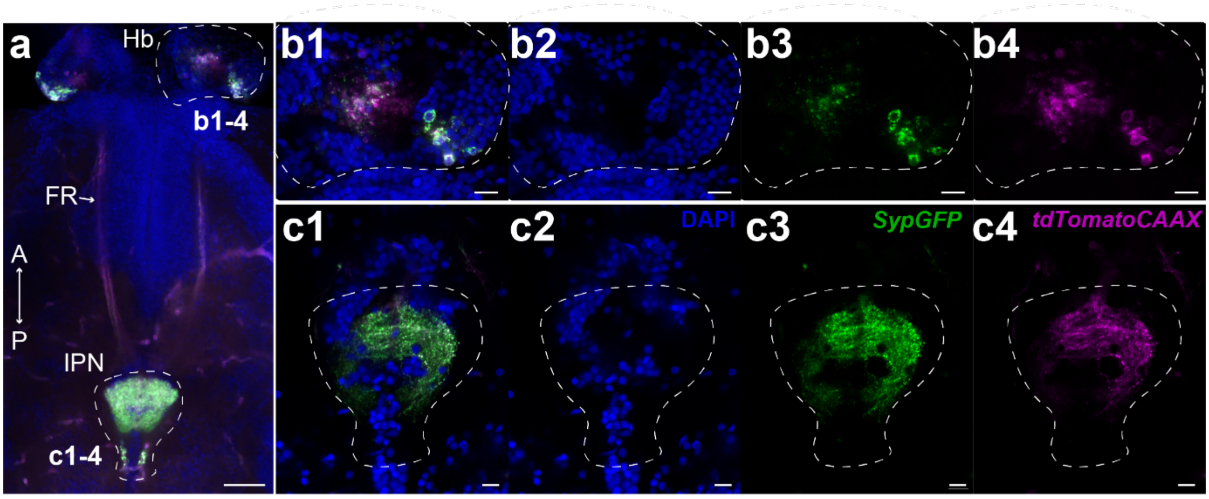

Supplementary Figure 8: Dorsal habenula neurons express presynaptic marker *Synaptophysin–GFP* both in axon terminals in the interpenducular nucleus as well as their processes in the habenula. Confocal images of *Tg(narp:GAL4VP16;UAS:Syp–GFP-T2A-tdTomato-CAAX)* juvenile zebrafish fixed, cleared and DAPI stained (example fish from n=5. Colors represent DAPI (blue), *Synaptophysin–GFP* (SypGFP, green) and *tdTomatoCAAX* (magenta). (**a**) Confocal image of the habenula and IPN merge of three channels. Dotted area indicated the habenula zoomed in **b1-4**, the interpenducular nucleus in **c1-4** and white arrow indicates the fasciculus retroflexus (FR). (**b**) Zoomed habenula images (delineated with dotted line) from A. Merged (**b1**), DAPI (**b2**, blue), *tdTomatoCAAX* (**b3**, magenta), *Synaptophysin–GFP* (**b4**, green). (**c**) Zoomed IPN images (delineated with dotted line) from A. Merged (**c1**), DAPI (**c2**, blue), *tdTomatoCAAX* (**c3**, magenta), *Synaptophysin–GFP* (**c4**, green). Scale bar represents 50 μm (**a**) or 10 μm. (**b-c**). Hb: habenula, IPN: interpenducular nucleus, FR: fasciculus retroflexus.

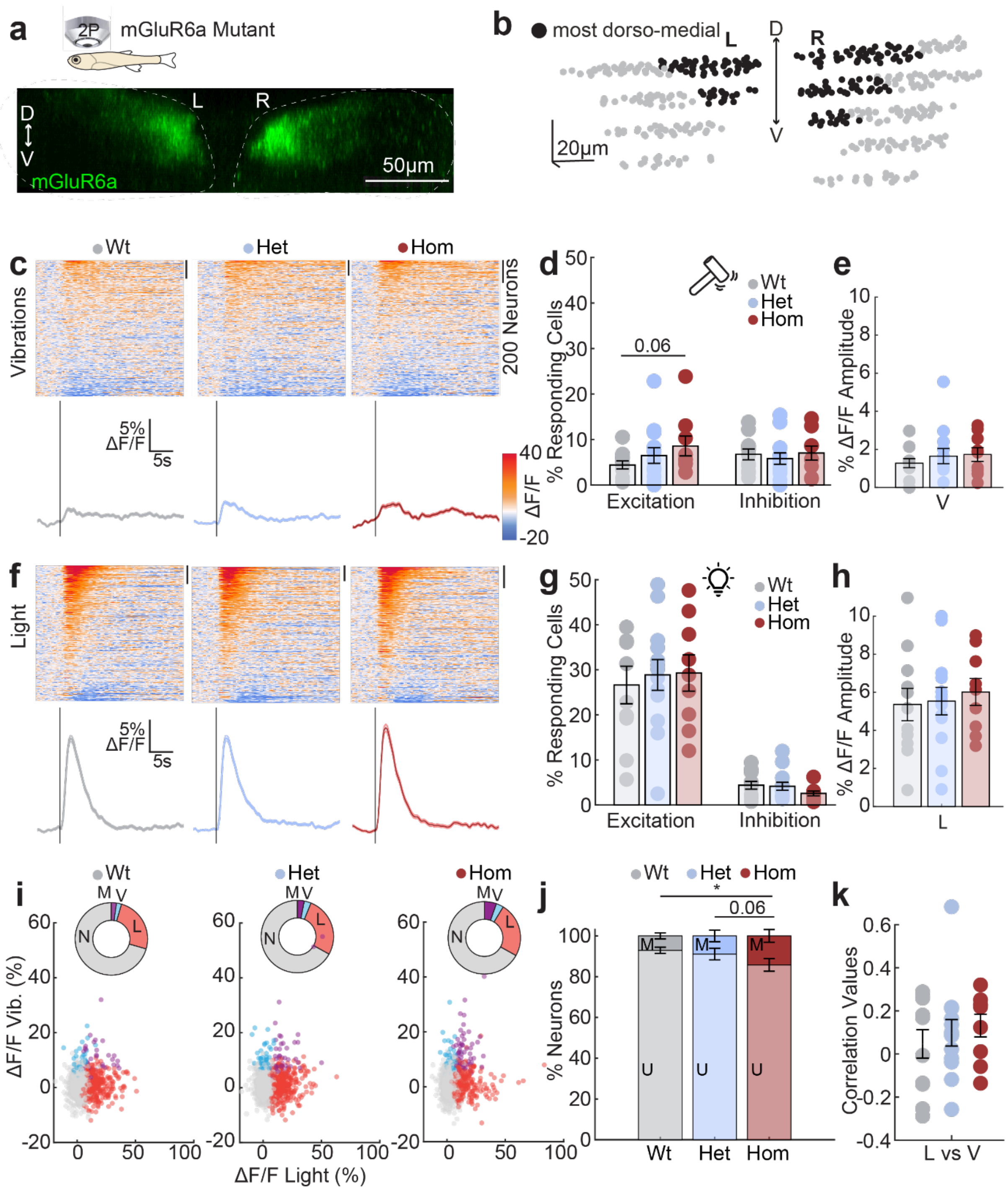

Supplementary Figure 9: Sensory responses of habenular neurons in mGluR6a mutants are altered. (**a**) Coronal confocal image of habenula that was stained by hybridization chain reaction for mGluR6a RNA expression (green). Borders of the habenula are delineated with white dotted lines. Scale bar represents 50 μm. (**b**) 3D-representative example of a Wt fish where the dorsomedial habenula cells (black) show similar location as compared to the mGluR6a expression pattern seen in A. (**c**,**f**) Heatmaps represent the time courses of habenular calcium signals (ΔF/F) in responses to vibrations (**c**) or light (**f**), in mGluR6a mutants expressing *Tg(elavl3:GCaMP6s* Left: Wt (n=1454 neurons, 11 fish), middle:

Het (n=1726 neurons, n=13 fish) or right: Hom (n=1182 neurons, n=9 fish) are sorted according to their mean activity in the response period (10s after stimulus onset). Warm colors indicate excitation, cold colors represent inhibition. Average traces of habenular responses are below each heatmap. Stimulus onset is indicated by a line. Shades represents SEM. (**d, g**) Percentage of excited (2 STD above baseline) or inhibited (1 STD below baseline) habenula neurons wildtype (grey), heterezygous (blue) or homozygous (red) fish in response to mechanical vibrations (**d**) or light (**g**) stimulation. Note more neurons in the homozygous fish respond to vibrations compared to Wt and Het. (Wt n=11, Het n=13, Hom n=9, Wt vs Hom p=0.0643, rest is n.s., one-sided Wilcoxon rank sum test). (**e,h**) Average ΔF/F response amplitude (%) during vibration (**e**) and light (**h**) in all habenular neurons in each fish. (Wt n=11, Het n=13, Hom n=9, p is n.s., one-sided Wilcoxon rank sum test). (**i**) Average response amplitude of individual habenula neurons to mechanical vibration (blue), light (red) or both (magenta) for control and CPPG injected fish. Donut chart represents the fraction of habenula neurons and their response type (2 STD above baseline). N: non-responding, V: only vibrations, L: only light, M: both vibrations and light. (**j**) Percentage of unimodal (U) habenular neurons that respond exclusively to either light (L) or vibrations (V) versus multimodal (M) neurons responding to both light and vibrations. Significantly less habenula neurons in the homozygous fish are selective for one of the two stimulus modalities (unimodal) compared to the wildtype fish, but instead more neurons are multimodal (Wt n=11, Het n=13, Hom n=9, Wt vs Hom *p=0.0286, Het vs Hom p=0.0578, rest is n.s., one-sided Wilcoxon rank sum test) (**k**) Pearson's correlation of multi-neuronal response vectors in the habenula for mechanical vibrations and light. Sensory responses in the homozygous fish are more correlated and hence more similar to each other (Wt n=11, Het n=13, Hom n=9, p is n.s., one-sided Wilcoxon rank sum test). P-values close to the significance threshold are reported as numbers in the figure. Error bars represent mean +/-SEM. Scattered dots represent individual fish. Source data are provided as a Source Data file.

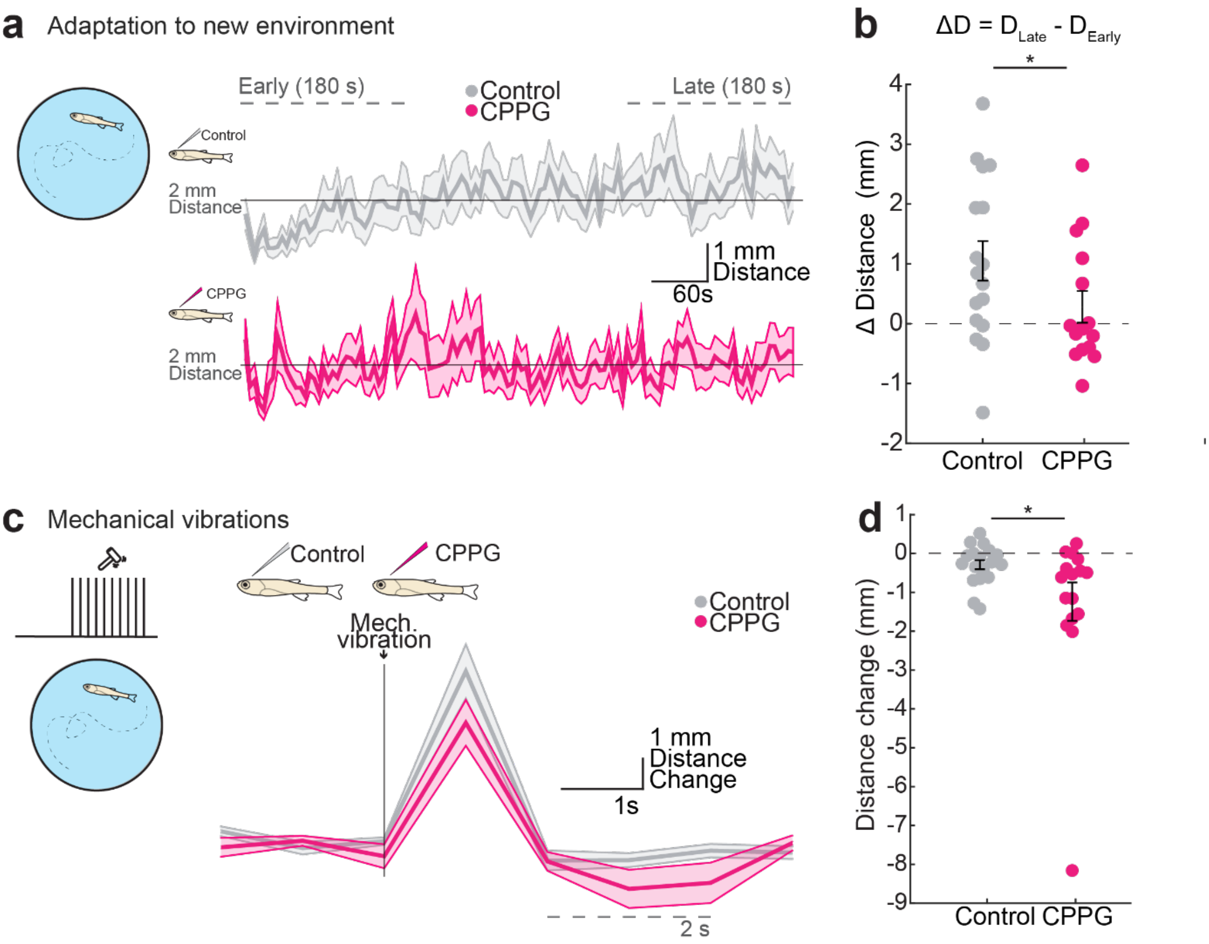

Supplementary Figure 10: Ventricular CPPG-injection enhances defensive behaviors in juvenile zebrafish. (**a**) Adaptation to a new environment in the first 10 minutes after the introduction to the behavioral arena. Average distance swam is plotted for control (grey, upper) and CPPG(5mM)-injected (pink, lower) separately for the two groups. The grey dashed line indicates the early and late period (180 s) that were used for statistical comparisons in **b**. Black horizontal line indicates the 2 mm swim distance value. Shadow represents the +/-SEM. (**b**) Difference in average distance swam (ΔD) between the early and late period (indicated in (A)) for control (grey) and CPPG-injected (pink) fish. Note that the CPPG-injected fish show a significantly lower change in distance compared to the control fish. (Control n=18 fish, CPPG n=17 fish, *p=0.0100, one-sided Wilcoxon rank sum test). (**c**) Mechanical vibrations are delivered to freely-swimming juvenile zebrafish in a horizontal tank after control- or CPPG-injection. Average changes in swimming distances (mm) evoked by mechanical vibrations (onset indicated by grey line and small arrow) are plotted. Grey dotted line indicates the sustained decreased swimming rate period (2 s) evoked by mechanical vibrations that was used for statistical comparisons in **d**. Shadow represents the SEM. (**d**) CPPG-injected fish show a sustained decrease in swimming rate compared to control-injected fish. (Control n=18,

CPPG n=16, *p= 0.0184, one-sided Wilcoxon rank sum test). Error bars represent mean +/- SEM. Scattered dots represent individual fish. Source data are provided as a Source Data file.
